# GLD-Net: Deep Learning to Detect DDoS Attack via Topological and Traffic Feature Fusion

**DOI:** 10.1155/2022/4611331

**Published:** 2022-08-16

**Authors:** Wei Guo, Han Qiu, Zimian Liu, Junhu Zhu, Qingxian Wang

**Affiliations:** State Key Laboratory of Mathematical Engineering and Advanced Computing, Zhengzhou 450002, China

## Abstract

Distributed denial of service (DDoS) attacks are the most common means of cyberattacks against infrastructure, and detection is the first step in combating them. The current DDoS detection mainly uses the improvement or fusion of machine learning and deep learning methods to improve classification performance. However, most classifiers are trained with statistical flow features as input, ignoring topological connection changes. This one-sidedness affects the detection accuracy and cannot provide a basis for the distribution of attack sources for defense deployment. In this study, we propose a topological and flow feature-based deep learning method (GLD-Net), which simultaneously extracts flow and topological features from time-series flow data and exploits graph attention network (GAT) to mine correlations between non-Euclidean features to fuse flow and topological features. The long short-term memory (LSTM) network connected behind GAT obtains the node neighborhood relationship, and the fully connected layer is utilized to achieve feature dimension reduction and traffic type mapping. Experiments on the NSL-KDD2009 and CIC-IDS2017 datasets show that the detection accuracy of the GLD-Net method for two classifications (normal and DDoS flow) and three classifications (normal, fast DDoS flow, and slow DDoS flow) reaches 0.993 and 0.942, respectively. Compared with the existing DDoS attack detection methods, its average improvement is 0.11 and 0.081, respectively. In addition, the correlation coefficient between the detection accuracy of attack flow and the four source distribution indicators ranges from 0.7 to 0.83, which lays a foundation for the inference of attack source distribution. Notably, we are the first to fuse topology and flow features and achieve high-performance DDoS attack intrusion detection through graph-style neural networks. This study has important implications for related research and development of network security systems in other fields.

## 1. Introduction

Popular industries such as shopping, education, finance, government affairs disclosure, and communications connect core services, such as payments, instant messaging, and big data analysis, to the Internet in real time for user access. Due to these services' vulnerability and high value, attacks on infrastructures that provide these services are favored by hackers. One of the most common attacks to block these services is the DDoS attack [1]. How to deal with DDoS attacks to ensure network smoothness has become a research hotspot [2].

The traditional defense strategy assumes that the attack topology is a static point-to-point model whose topology remains unchanged during the attack [3, 4]. Under this premise, DDoS detection is mainly realized through changes in traffic size, and the corresponding single-point defense is relatively simple [5]. In 2022, Israel's network providers were hit by a large DDoS attack from abroad, paralyzing the website of the Interior Ministry for hours. The same year, the Ukrainian government suffered repeated DDoS attacks from Russia and Belarus, forcing multiple portals to shut down to avoid losses. The network situation has changed as DDoS attacks shift from individual behaviors to confrontations between countries. Except for the increase in the attack traffic, the range of attack sources continues to expand; the flow topology evolves during the attack. Conventional single-point defense cannot cope with these changes, and multisource protection requires attack source location. However, previous detections cannot identify the attack distribution, thus impossible to precise defense. Therefore, we need a detection method that can determine the attack distribution through topology changes to support further attack tracing and defense deployment.

DDoS detection aims to distinguish attack traffic from legitimate traffic. According to the different fields of mathematics, the current mainstream DDoS detection methods can be divided into three categories: statistics, machine learning, and deep learning. The statistical method uses measures such as entropy to evaluate the traffic distribution's change. It is simple and requires no additional hardware support. However, its detection effect depends on thresholds, which researchers usually give directly [6]. This subjective assignment lacks an objective basis, affecting the reliability of results. Machine learning classifies network traffic through classifiers designed based on selected features. Due to modeling using features, it exhibits excellent flexibility. However, reliance on feature engineering makes it less adaptable in the face of complex real network traffic. In addition, traditional machine learning belongs to shallow learning, making it difficult to learn deep relationships. Thus, its accuracy is usually less than 90%. Deep learning utilizes multilayer neural networks to learn the inherent laws of network traffic. The feature extraction is contained in the neural network structure without additional processing. Besides, multilayer neural networks can mine deep information, making up for the defects of shallow learning. DDoS detection based on deep learning has high accuracy and efficiency. For different requirements and problems, many related research studies are emerging [7]. These studies usually improve performance by improving or fusing network architectures. For example, convolutional neural network (CNN) and recurrent neural network (RNN) are used to process the relationship between features in time and space [8], RNN and automatic codec are combined to improve the detection sensitivity on SDN [9], and adaptive transfer learning is introduced to achieve small sample detection [10]. These methods achieve good performance by exploiting the efficient information in the input as much as possible through elaborate devised architecture and parameters.

DDoS attacks have two notable characteristics: (1) from a spatial perspective, heavy traffic in the short term changes the distribution of adjacent upstream nodes of the victim host [11]. (2) From a time perspective, the prolonged blocking makes the limited attack nodes have multiple attack behaviors on the target [12]. These two intrinsic peculiarities of DDoS attacks make the network topology before and after the attack significantly different. Therefore, in addition to traffic characteristics, DDoS attacks can also be detected based on the topology changes [13]. This difference is implicit in the evolution of the topology structure, which traffic statistics cannot depict. Introducing topology changes can improve detection accuracy and help analyze the distribution of attack sources. The data (such as graph) considering topological connection are non-Euclidean data. Sample points (nodes) have different numbers of neighbor points, and edges depict their interdependence. However, traditional deep learning requires Euclidean data as input to extract features. For example, CNN needs the sample to be regular and independent. RNN demands the data to be a one-dimensional real vector. The linear input cannot deal with topological relationships. Graph attention network (GAT) is a powerful analysis tool for graph data [14]. It incorporates the attention mechanism into the graph neural network and captures associations through neighborhoods. Further, the attention mechanism assigns different weights to adjacent nodes, improving feature sensitivity. In this study, we treat topology as graph data. In particular, edge attributes denote traffic features, and node attributes indicate topological features. Therefore, GAT can simultaneously analyze traffic and topological features with the graph as input. To our knowledge, we are the first to achieve DDoS attack detection using graph-style deep learning. The main contributions of this study are as follows:

1. The proposed dynamic topology construction algorithm integrates topology and flow features into node or edge attributes.
2. GAT is used to mine topology change patterns and train classifiers.
3. Compared with other methods, the deep learning method integrating topology and traffic features achieves higher accuracy in both two classifications and three classifications of DDoS traffic.
4. The proposed detection method supports estimating the distribution of attack traffic sources.

The rest of this study is organized as follows. In Section 2, we discuss research related to DDoS detection.  Section 3 describes the details of the proposed method in terms of feature extraction and deep learning architecture.  Section 4 designs the experiments and analyzes the results. We summarize this research in Section 5. Finally, the shortcomings and future research are pointed out in Section 6.

## 2. Related Work

In recent years, DDoS detection research mainly acquires traffic features containing attack-specific information through feature acquisition [15]. Then, characteristics are analyzed based on different theories or tools to discover traffic classification patterns [16]. Section 2 describes current DDoS feature acquisition methods and summarizes three mainstream DDoS detection methods: statistics, machine learning, and deep learning.

### 2.1. Feature Acquisition

Valid feature input is critical to traffic classification performance since it determines the valuable information contained in samples. There are two main methods for feature acquisition: output features using generator tools (such as CICFlowMeter) [17, 18] and custom features based on subjective experience [19, 20]. The former applies public datasets or traffic extraction tools to obtain features, while the latter designs corresponding features according to application requirements. In 2017, Yuan et al. [17] extracted 20 network traffic fields from the ISCX2012 dataset for *DeepDefense* detection model training. This method is simple and avoids complex statistical feature calculations. In 2018, Idhammad et al. [18] reduced the feature dimension of datasets such as UNSW-NB15 based on collaborative clustering. Then, simplified datasets were used to test machine learning methods' classification performance. The results show that this method effectively reduces the false-positive rate. In 2018, Doshi et al. [19] extracted three stateless and two stateful features through network packet behavior, which showed high accuracy in IoT traffic detection. In 2019, De Lima Filho et al. [20] utilized 25 IPv4 variables to design 33 signature features suitable for IP, UDP, and TCP, which improved the sensitivity of online DDoS detection. In 2022, Chouhan et al. [21] defined the seven most relevant features for real-time traffic detection. They extracted these features from switch statistics based on the Ryu controller module, reducing the identification delay of the classifier.

The above methods propose efficient feature acquisition strategies. Nevertheless, these flow feature extraction ways lack the characterization of the topology. Therefore, it is needed to define topological features and give corresponding acquisition methods.

### 2.2. Statistical Method

Statistical methods use numerical distribution to differentiate traffic. In 2017, Hoque et al. [22] proposed a new correlation indicator NaHiD based on standard deviation and mean. Experimental results show that this measure is more robust and sensitive to state changes than traditional metrics. In 2022, Tsobdjou et al. [23] raised a dynamic entropy threshold algorithm based on Chebyshev inequality. Comparative experiments indicate that this method can better adapt to varied online environments than static thresholds. The same year, Ahalawat et al. [24] proposed a Renyi entropy DDoS attack detection technique based on the packet drop strategy. It can evaluate the probability distribution of flow fluctuations and achieve better results than the Shannon entropy.

These methods analyze the numerical fluctuation of flow from a macro-view. However, their application scope is narrow due to the lack of fine-grained characterization. Thus, statistical methods are usually not used alone for comprehensive evaluation.

### 2.3. Machine Learning

Machine learning can automatically learn feature patterns and create classifiers. In 2019, Gu et al. [25] proposed the DDoS detection algorithm SKM-HFS. Weighted K-means analysis balances the number of samples and accuracy, and the density clustering center algorithm optimizes the extreme values. The results show that this method performs best when choosing TOPSIS as the evaluation factor. In 2020, Pande et al. [26] utilized the random forest algorithm to distinguish between normal and attack samples and used the WEKA tool to detect DDoS attack ping of death. Experiments on NSL-KDD indicate that random forest achieves the highest accuracy of 99.76% on specific attacks. In 2021, Cvitic et al. [27] understand DDoS detection as a multi-device classification problem and distinguish traffic generated by different IoT devices through a logical model tree. A comparison of four typical devices shows that the logical model tree can better identify DDoS traffic from IoT devices. In 2022, Kumar et al. [28] designed the recursive feature elimination method RFE. It is also combined with the random forest algorithm to train the classifier. Experiments show that this method can cope with fast detection under large network traffic.

The above methods extract relevant information from the traffic details. However, they rely heavily on feature engineering and have low performance in the face of large samples. Hence, we need to find a more efficient detection model.

### 2.4. Deep Learning

Deep learning applies a multilayer neural network to obtain the correlation between input and output. In 2019, Liang and Znati [29] employed LSTM in a DDoS detection framework. LSTM captures the implicit sequence representation in the input vector through three gating units. This method can learn flow-level modes, avoiding expensive and error-prone feature engineering. In 2020, Doriguzzi-Corin et al. [30] proposed LUCID, a lightweight DDoS detection system that utilizes one-dimensional CNN to reduce computational load. Experiments on ISCX2021, CIC-IDS2017, and CSE-CIC2018 datasets show that LUCID has a 40x reduction in processing time compared with other deep learning methods, so it is suitable for detection under limited resources. In 2021, Cil et al. [31] built a traffic classification model based on the deep neural network. Its structure contains feature extraction, and training can be completed with only three fully connected layers. Experiments on CIC-DDoS2019 show that the model has an accuracy of 95%. In 2022, Boonchai et al. [32] implemented two DDoS detection models using the DNN architecture and autoencoder, respectively, and verified the attack recognition ability of the models through the CIC-DDoS2019 dataset with an accuracy rate of 87% and 91.9%, respectively.

A single method is challenging to meet diverse DDoS detection needs. Therefore, many scholars extend the applicability through method mixing. In 2019, Pektaş and Acarman [8] extracted five statistical features: duration, bytes, packets, periodicity, and states through network traffic summary and mined semantic information in the feature sequence through CNN and RNN. The accuracy of this method reaches 99.1%, significantly higher than a single network. In 2020, Wang and Liu [33] employed information entropy and deep learning to detect DDoS attacks in SDN. First, IP entropy identifies malicious traffic routers, and then, CNN classifies packet-level traffic. This method achieves 98.98% accuracy and also reduces training time. In 2020, Elsayed et al. [9] proposed DDoSNet, an intrusion detection system for SDN. This system combines RNN and autoencoder. RNNs capture sequence relationships, and autoencoders detect small perturbations. Compared with baseline methods such as decision tree, random forest, and support vector machine, DDoSNet is more stable and achieves an accuracy of 99%. In 2021, Shieh et al. [34] built a DDoS unknown traffic discovery model BI-LSTM-GMM. It consists of bidirectional LSTM (BI-LSTM) and Gaussian mixture model (GMM). GMM labels the unknown traffic and adds it to the new input of BI-LSTM. Experiments show that this method can identify unknown attacks through reinforcement learning. In 2022, Almaraz-Rivera et al. [35] designed a new intrusion detection system based on machine learning and deep learning models to solve the unbalanced detection of DDoS attack categories. It combines decision trees and multilayer perceptrons to test binary classification performance on different datasets, avoiding data and fragmentation effects.

Besides binary classification, multi-classification studies that ease defense deployment are also emerging. In 2019, Toupas et al. [36] utilized stacked fully connected layers for intrusion detection. Experiments show that this method can better learn the difference between fast and slow DDoS flows with an accuracy of 95.62%. In 2020, Alzahrani et al. [37] proposed FastGRNN, a DDoS multi-classification method for IoT, which reduces training complexity by adding residual to hidden states. It achieves 1:5 optimization of detection time and training time to adapt to real-time detection. In 2020, Hussain et al. [38] used ResNet for complex traffic detection. They convert traffic into a three-channel format and analyze it through ResNet. This method achieves an accuracy of 87% in distinguishing normal flow, fast DDoS flow, and slow DDoS flow and an increase of 9% compared with other methods. In 2022, Rusyaidi et al. [39] designed a high-precision DDoS attack detection system based on DNN and LSTM. It achieved an accuracy of 97.37% on the NSL-KDD dataset and excellent performance in identifying 22 traffic types.

With the in-depth development of deep learning, many researchers also apply new architectures to optimize DDoS detection performance. In 2020, He et al. [10] employed transfer learning for small-sample DDoS detection. They also define the transfer ability to evaluate different networks and select the best network structure and parameters. This method improves the detection accuracy on small samples by 20.8%, which can effectively cope with training degradation. In 2021, Novase et al. [40] utilized generative adversarial network (GAN) to detect DDoS adversarial attacks. It improves system robustness through adversarial training and uses IP entropy to analyze continuous traffic for real-time monitoring. This method shows strong adaptability in detecting adversarial attacks. In 2022, Doriguzzi-Corin and Siracusa [41] proposed an adaptive mechanism for DDoS attack detection based on federated learning, FLAD. It updated federated learning to solve the integration problem in dynamic security confrontation, monitoring the status locally without interaction. The experimental results verified the efficiency and performance of the method.

Deep learning has shown advantages in different detection requirements. However, it can only process traffic characteristics and not extract topology information. Thus, we need to find a new way to consider both features to improve detection accuracy and lay a basis for attack source localization.

## 3. Methodology

**Figure 1:**
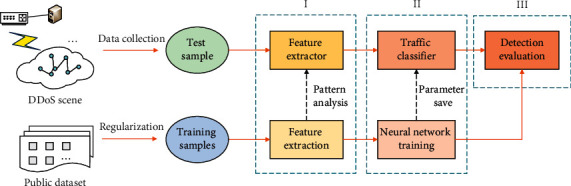
Framework of DDoS attack detection system based on deep learning.

This section details the procedure and implementation of deep learning detection based on topological and flow features. As shown in Figure 1, our proposed DDoS detection system has three main parts. The first part is the extraction module. It is responsible for extracting features from public datasets or actual scenes and transforming samples into graph data consisting of nodes and edges. The second part is the training module, which builds a classification model that can mine deep-level information from samples. The input is sample data, the output is label type, and parameters are optimized during training. The third part is the evaluation module, which compares detection effects under different hyperparameters to select the optimal configuration.

When the above stages are completed, the pattern analysis for feature extraction is saved as an extractor, and the trained neural network is preserved as a classifier. Then, real traffic can be quickly classified by running through these processing parts only once without retraining.

### 3.1. Feature Extraction

**Figure 2:**
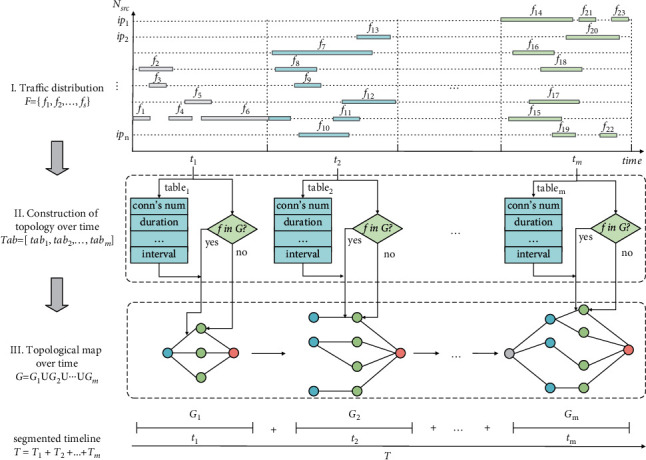
An example of topological feature extraction and topological map construction process based on time series.

Building a topology diagram is the core of topology feature extraction. It maps from raw traffic data to dynamic topology plots that evolve; an example is shown in Figure 2. In particular, *f*_*i*_ denotes the flow's distribution originating from the corresponding *ip*_*n*_, *G*_*m*_ denotes the subgraph under time slice *t*_*m*_, and the indicators in the feature table denote extracted samples. Besides, *F*, *Tab*, *G*, and *T* represent the set of flow distribution {*f*_1_, *f*_2_, ⋯, *f*_*s*_}, feature table [*tab*_1_, *tab*_2_, ⋯, *tab*_*m*_], topology map *G*_1_ ∪ *G*_2_ ∪ ⋯∪*G*_*m*_, and time slice *T*_1_+*T*_2_+⋯+*T*_*m*_, respectively. In Figure 2, there are two major stages. The first stage realizes the transformation from traffic data to node or edge features. The traffic records of different source IPs are divided according to the time unit. Then, the time slice proportions of features are formed into [IP-Feature] pairs and saved in the feature table. The second stage builds the connection graph, adds attributes to nodes or edges according to the feature table, and decides whether to add based on the flow that exists or not in the topology. The final topological feature map *G*=*G*_1_ ∪ *G*_2_ ∪ ⋯∪*G*_*m*_ on *m* sub-time segments is obtained when the addition is finished.

In Figure 2, the feature table determines the attributes of edges and nodes. Besides statistical features, we also add connection state, packet marker, and centrality features. The attack pattern implied in the connection state sequence can distinguish attack phases [8]. For example, the target maintains many half-open connections in a SYN flood attack, making the state list of long LISTENs. The normal communication state is composed of LISTEN, ESTABLISHED, and CLOSED. In this case, the proportion of LISTEN differs from that of SYN attack. Therefore, state sequences reflecting this divergence can be used for traffic classification. Packet flags reveal the attacker's malicious attack intention. For example, regular data packets must be queued in the buffer before parsing, while numerous URG flags set to 1 increase the processing priority, thus enabling fast attacks. Hence, the packet tampering details that macroscopic features cannot describe are hidden in the packet marking sequence, thus detecting the attack. We choose the degree and betweenness centrality based on the understanding that destructive attackers usually control more agents to execute attacks, and targets are generally critical nodes [42]. Then, these two centralities can capture the attack preference to realize attack detection. In conclusion, we extract multiple node or edge features for attack detection, addressing the one-sidedness of the training data.

We extract features from ICMP, UDP, and TCP, respectively. The features of protocols except TCP are the same. For clarity, we take TCP features as an example to illustrate the attributes of the extracted features, as shown in Table 1. Among them, *t*_s_ represents an arbitrary time field.

Table 1 includes eight edge and two node features. In particular, the edge feature depicts the traffic distribution through the edge in period *t*_s_; the node feature describes the spatial distribution of adjacent nodes within *t*_s_. URG and ECE flags are extended to lists to preserve the time-varying properties. We also introduce degree and betweenness centrality to characterize topology changes. The meaning and acquisition of the features in Table 1 are described below.

Among edge features, except “connection number,” “connection states,” “URG flag,” and “ECE flag,” the other four can be calculated by statistical formula. Notably, the standard deviation measures the discrete distribution of samples. When the number of samples with the same IP and protocol in *t*_s_ is 3, regular flows are much more than attack flows, making the training challenging to converge. So, when the number of samples is not less than 3, the standard deviation has practical significance. In addition, in subsequent experiments, we found that the detection efficiency and accuracy are balanced when the number of samples is not less than 4. Therefore, we only consider four or more identical protocol connections established between the same node pair as actual training data.

Connection number and connection state are two macro-edge features. The former reflects the frequency of establishing connections between nodes; the latter reflects the continuous change in the protocol state. For example, CLOSED means all active links are closed; LISTEN signifies waiting for new requests; and ESTABLISHED means the connection is established. These states can be obtained through traffic analysis tools like TShark or CICFlowMeter [43]. However, the acquired elements are of type string and hard to use for training directly. We use one hot to convert state sequences into real vectors to simplify computation [44]. In particular, zero indicates that the state is not enabled, and one denotes that the state is activated.

“URG flag” and “ECE flag” record the state sequence of consecutive packets. These two flags represent unexpected events during the sending of traffic. In particular, a URG of 1 indicates that the current data packet is prioritized and should be processed without queuing; an ECE of 1 indicates congestion, and the sending window decreases. The abnormal state to one is set, and the normal state is set to zero; then, the state sequence is a list of zeros and ones. This binary list can be used for training directly without encoding.

Two node features, degree and betweenness centrality, characterize the connectivity properties of the neighborhood. Degree centrality uses the number of adjacent nodes to denote the node importance. Let *Len* be a function of solving the number of non-repetitive elements; *N*_*neighbor*_ represents the number of neighboring nodes of node *N*; 〈*N*_*src*_^*s*^, *N*_*des*_^*s*^〉 indicate flow *s*. Then, degree centrality is formulated as follows:

(1) $$\begin{array}{c} N_{degree}=\frac{N_{neighbor}}{Len\left(\left(N_{src}^{1},\cdots ,N_{src}^{s}\right)\cup \left(N_{des}^{1},\cdots ,N_{des}^{s}\right)\right)-1}. \end{array}$$

Betweenness centrality measures the node importance by the ratio of shortest paths' number through a node. Let *Path*(*N*) denote the number of shortest paths containing node *N,* and *Path*(*src*_*i*_, *dst*_*j*_) denote the number of shortest paths between *src*_*i*_ and *dst*_*j*_. Then, the formula of betweenness centrality is as follows:

(2) $$\begin{array}{c} N_{between}=\frac{Path\left(N\right)}{\sum_{i,j}Path\left(src_{i},dst_{j}\right)}. \end{array}$$

We also show the construction process of the DDoS topology map through pseudo-code, as shown in Algorithm 1.

Algorithm 1 can be divided into three main stages, namely, the initial assignment stage (1∼2), configuration stage (3∼11), and mapping stage (12∼21). The graph structure is initialized in the initial assignment phase, and necessary parameters are set. Statistical features are calculated based on item durations' total and average in the configuration stage. In the final phase, nodes and directed edges are added to graph G, and features are attached to them. After the above steps are completed, the topology graph G is constructed.

To sum up, by extracting the features in Table 1 and constructing a dynamic topology graph according to Algorithm 1, the flow or topology attributes are included in the node or edge features. Till now, we have obtained structured data for training.

### 3.2. Architecture of Deep Learning Model

**Figure 3:**
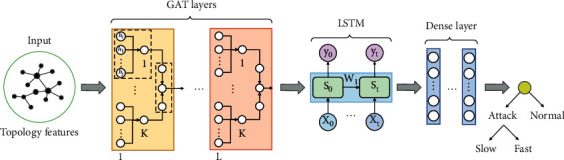
Overall architecture of the deep learning model GLD-Net.

This part introduces GLD-Net, a deep learning model capable of analyzing and fusing topology and flow features. Its structure is shown in Figure 3. This model has three main parts: the GAT layer, LSTM, and the fully connected layer (also known as the dense layer). Firstly, an L-layer GAT network is used to analyze the topological data. Its output is a spatial sequence over the neighborhood. Secondly, sequence relationships in the output are mined by LSTM. Finally, the dense layer reduces the feature dimension, and the softmax function limits the output size between zero and one. This value corresponds to the traffic label to achieve classification. In the following subsections, we will detail the processing method of each neural network and the information transfer within it. In particular, L and K in Figure 3 represent the number of attention mechanisms and the number of splice heads in multi-head attention, respectively. The detailed parameter functions and setting methods of the GAT, LSTM, and dense layer will be explained in each subsection.

#### 3.2.1. GAT Layer

**Figure 4:**
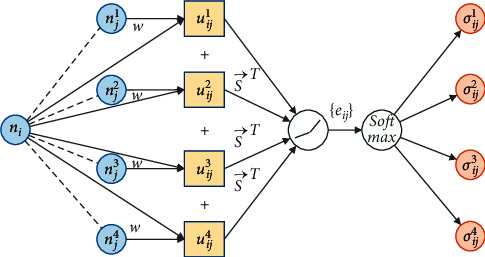
Information transfer process of the attention coefficient calculation in GAT.

The two basic units of GAT are attention coefficient calculation and information aggregation, as shown in the dotted box in Figure 3. The structure of the attention coefficient calculation is shown in Figure 4.

In Figure 4, we take four adjacent nodes *N*_*j*_=(*n*_*j*_^1^, *n*_*j*_^2^, *n*_*j*_^3^, *n*_*j*_^4^) of node *n*_*i*_ as an example to illustrate the information transfer progress in calculating the attention coefficient. Let *U*_*ij*_=(*u*_*ij*_^1^, *u*_*ij*_^2^, *u*_*ij*_^3^, *u*_*ij*_^4^), *ur*/*ij* be the intermediate variables obtained by splicing the initial node *n*_*i*_ and the adjacent node *n*_*j*_^*r*^ after feature enhancement *φ*, and Concat denote concatenation operation; that is,

(3) $$\begin{array}{c} \begin{array}{cc} u_{ij}^{r}=\mathrm{Concat}\left(\varphi \left(n_{i}\right),\varphi \left(n_{j}^{r}\right)\right), & r=\left[1,2,3,4\right] \end{array}. \end{array}$$

Let *w* be a trainable shared weight, and *φ*(*n*_*i*_) and *φ*(*n*_*j*_^*r*^) can be obtained by linear transformations, which are, respectively, expressed as follows:

(4) $$\begin{array}{c} \begin{array}{cc} \varphi \left(n_{i}\right)=w\cdot n_{i}, & \varphi \left(n_{j}^{r}\right)= \end{array}w\cdot n_{j}^{r}. \end{array}$$

A similarity coefficient *e*_*ij*_^*r*^ can be obtained by the inner product of the intermediate variable *u*_*ij*_^*r*^ and the trainable parameter vector $\overset{⟶}{s}$. In addition, the deviation of similarity coefficients is corrected by LeakyReLU. The negative axis slope of LeakyReLU retains negative values so that similarity coefficients do not suffer from the loss of negative information like ReLU. The formula of *e*_*ij*_^*r*^ is expressed as follows:

(5) $$\begin{array}{c} e_{ij}^{r}=\mathrm{LeakyReLU}\left(\langle {\overset{⟶}{s}}^{T},u_{ij}^{r}\rangle \right). \end{array}$$

*e*_*ij*_^*r*^ needs to be normalized on the interval [0, 1] to facilitate information aggregation. According to different transformation modes, normalization can be divided into linear methods, such as min-max [45] and Z-score [46], and nonlinear methods, such as softmax [47]. In particular, min-max only needs extremum and current values. Its calculation is simple but easily affected by individual points. Z-score utilizes comprehensive information and is less affected by outliers. However, the data must meet the normal distribution; otherwise, the output will be seriously distorted. The exponential calculation of softmax is a smooth derivative transformation that retains each value's influence and has no data distribution requirements. We choose the nonlinear function softmax as the normalization method to prevent the loss of complex information in the transformation process. Then, the attention coefficient *σ*_*ij*_^*r*^ can be calculated by the following formula:

(6) $$\begin{array}{c} \sigma_{ij}^{r}=\frac{\exp\left(e_{ij}^{r}\right)}{\sum_{k=1}^{4}\exp\left(e_{ij}^{k}\right)}. \end{array}$$

**Figure 5:**
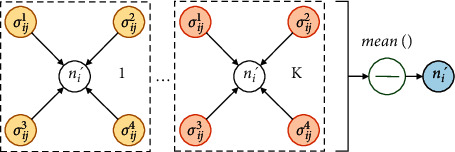
Flow mechanism of the information aggregation based on attention coefficients in GAT.

The attention coefficient *σ*_*ij*_^*r*^ contains the correlation between node *n*_*i*_ and neighbor node *n*_*j*_^*r*^. Then, the information aggregation based on neighborhood nodes can be realized with the attention coefficient. Its structure is shown in Figure 5.

In Figure 5, the new node feature *n*_*i*_′ can be calculated by the weighted sum of all neighbor node features *φ*(*n*_*j*_^*r*^) with *σ*_*ij*_^*r*^. The calculation formula is as follows:

(7) $$\begin{array}{c} n_{i}^{\prime }=\tau \left(\sum_{r=1}^{4}\sigma_{ij}^{r}\cdot \varphi \left(n_{j}^{r}\right)\right), \end{array}$$

where *τ* is the transformation function that maps the original vector space *ℝ*^*r*^ to a new vector space *ℝ*^*r*′^ centered at *n*_*i*_′. Due to errors, there may be offsets in a single calculation. Therefore, we use multi-head attention with parameter *K* to improve the robustness of the results. Commonly used methods of combining multiple attention include concatenation and averaging [14]. We choose arithmetic averaging as the synthesis algorithm for reduced dimensionality and higher efficiency. Then, the following formula is obtained:

(8) $$\begin{array}{c} n_{i}^{\prime }\left(K\right)=\frac{1}{K}\left(\tau \sum_{h=1}^{K}n_{i}^{\prime }\left(h\right)\right). \end{array}$$

Except for the learnable parameters *w* and $\overset{⟶}{s}$, other parameters, including the number of attention mechanisms L, the number of multiple heads K, and the gradient *θ* of LeakyReLU, are hyperparameters configured before training. Grassia et al. [48] pointed out that the size of L is related to the ability of information aggregation, and a single attention mechanism can learn node features up to 3 hops away. Thus, the bigger L is, the wider the range of information aggregation is. In datasets such as NSL-KDD2009, the number of IP hops of data packets does not exceed seven jumps [49], so L set to three can meet the requirements. K determines the learning perspective of relevant information. Efficiency and accuracy are balanced when K is set to 20 in subsequent experiments. *θ* affects the weight update rate, and its value should be adapted to the dataset size and learning depth. Combined with the tradeoff theory [50], *θ* is set to 0.3.

To sum up, we achieve local information aggregation of node features through GAT's feature transformation and multi-head attention mechanism. This study considers edges and nodes as entities of the same status. Edge features are merged into node features for unified processing to simplify computation.

#### 3.2.2. LSTM

After GAT training, the output vector *n*′ = {*n*_*i*_′, *i* ∈ *no*  *de*} is obtained. This vector contains the spatial sequence information of nodes in the neighborhood. Suppose it is directly poured into the classifier without processing. In that case, it will cause the loss of semantic information. Common network structures for processing sequence data are RNN and LSTM [51]. RNN uses memory units to retain historical data. Thus, the output is determined by the previous data and current input. However, due to the disappearance of the back propagation gradient, it is easy to cause short-term memory. LSTM enhances memory with gating units to learn relevant information in longer sequences. There are many adjacent attack nodes in DDoS attacks, making the distance between related data in the original data larger. Therefore, we choose LSTM to mine long sequence information. For clarification, we use the input *x*_*t*_ of LSTM at time *t* as an example to illustrate the information flow. In Figure 3, the memory cell *S*_t_ at time *t* comprises three gating units: input gate *I*(*t*), forgetting gate *F*(*t*), and output gate *O*(*t*). Assuming that the output of the memory cell at time *t*-1 is *c*_*t*−1_, then the calculation formulas of *I*(*t*), *F*(*t*), and *O*(*t*) at the next moment *t* are as follows:

(9) $$\begin{array}{c} \begin{array}{c} I\left(t\right)=sigmoid\left(W_{i}^{t}\cdot concat\left(c_{t-1},x_{t}\right)+b_{i}^{t}\right)\\ F\left(t\right)=sigmoid\left(W_{f}^{t}\cdot concat\left(c_{t-1},x_{t}\right)+b_{f}^{t}\right)\\ O\left(t\right)=sigmoid\left(W_{o}^{t}\cdot concat\left(c_{t-1},x_{t}\right)+b_{o}^{t}\right) \end{array}, \end{array}$$

where *W*^*t*^ and *b*^*t*^ represent the weight and bias of transformation at time *t*, respectively. The sigmoid function controls the values of *I*(*t*), *F*(*t*), and *O*(*t*) to fall within the interval [0,1]. In particular, one means all the information flow is passed, and zero means the information flow is blocked. Let $\tilde{B}\left(t\right)$ be the data to be processed, and its calculation formula is as follows:

(10) $$\begin{array}{c} \tilde{B}\left(t\right)=\tanh\left(W_{e}^{t}\cdot concat\left(c_{t-1},x_{t}\right)+b_{e}^{t}\right), \end{array}$$

where *W*_*e*_^*t*^ and *b*_*e*_^*t*^ are the parameters of memory cell state transition. Assume the intermediate state of the memory cell at time *t*-1 is *B*_*t*−1_. The recording of $\tilde{B}\left(t\right)$ and the forgetting of *B*_*t*−1_ are controlled by *I*(*t*) and *F*(*t*), respectively. Let ⊗ denote the defined gating transformation; then, at the next moment, the updated intermediate state *B*_*t*_ can be expressed as follows:

(11) $$\begin{array}{c} B_{t}=F\left(t\right)⊗B_{t-1}+I\left(t\right)⊗\tilde{B}\left(t\right). \end{array}$$

The output gate *O*(*t*) controls the actual information passing through the intermediate state *B*_*t*_. Then, the formula to obtain the final output *c*_*t*_ is as follows:

(12) $$\begin{array}{c} c_{t}=O\left(t\right)⊗\tanh\left(B_{t}\right). \end{array}$$

In addition to the learnable weight *W* and bias *b*, the calculation of *c*_*t*_ has three key parameters: the input vector dimension, the state dimension of the intermediate layer, and the number of memory cell layers. In particular, the input vector dimension is consistent with the received data *n*′. The middle layer's state dimension determines memory cells' learning ability. It is set to 32 to cover as many patterns as possible. The internal recursive structure of LSTM makes nonparallel operations more complicated. Noticeably, the excellent extraction ability of LSTM makes it unnecessary to stack too many layers in practical applications. For example, Google Translate only requires no more than eight layers to complete the vast majority of bidirectional translation tasks [52]. In this study, when the number of memory cell layers is set to 3, the correlation extraction of DDoS data can be satisfied.

#### 3.2.3. Dense Layer

The previous chapter realized the fusion of sequence information. Then, in this part, the final evaluation value will be obtained based on information aggregation. In Figure 3, LSTM is followed by fully connected layers, constituting a classifier with the dropout layer and activation function. GAT and LSTM map the DDoS raw sample to the feature space. Then, the fully connected layer maps the learned feature representation to the DDoS label space. The dense layers are set to 3 to learn nonlinear correlation [53]. The number of neurons in each layer is 128, 64, and 32, considering the running efficiency and learning ability. We also add dropout layers after the first and second layers to avoid overfitting. In testing, the removal probability was set to 0.3 to improve the model's generalization ability. Distinguishing the attack type (slow or fast) is a multi-classification problem. Softmax is selected to assign probabilities between 0 and 1 for different input samples. The formula is as follows:

(13) $$\begin{array}{c} P_{i}=\mathrm{softmax}\left(c_{i}\right)=\frac{e^{c_{i}}}{\sum_{i}e^{c_{i}}}. \end{array}$$

This model belongs to supervised learning. Labels allow the model to use the feedback value of the cross-entropy loss function to correct errors during the back propagation. The weights and biases are updated layer by layer to approximate the expectation. Training ends when all iterations are over. Training is done multiple times, and the best performing parameters are saved for fast classification.

## 4. Experiment

In this section, we elaborate on the implementation and evaluation details of the proposed method. First, running environments are illustrated to enhance the reproducibility of results. Secondly, training datasets are selected, and data preprocessing is given. Then, measures including accuracy, recall, precision, and F1-score are used to evaluate the effectiveness of detection methods. Finally, compared with baselines and state-of-the-art techniques, the performance of the proposed method is verified, and its efficiency is examined. Further, the correlation between the detection value and the source distribution is also analyzed.

### 4.1. Running Environment

The experiments were run on a Windows 10 workstation with Intel Core i7-12700H 4.7 GHz processor, 32 GB RAM, 512 GB SSD, and NVIDIA RTX 3060 graphics card. The GLD-Net model uses Python 3.5 as the programming language and adopts Keras as the deep learning framework to improve portability. Keras provides structured modules and connects to the GPU for acceleration via the backend engine TensorFlow's cuDNN library. Additionally, libraries such as Pickle, NumPy, and SciPy are loaded to enhance the efficiency of algorithms.

### 4.2. Datasets

**Figure 6:**
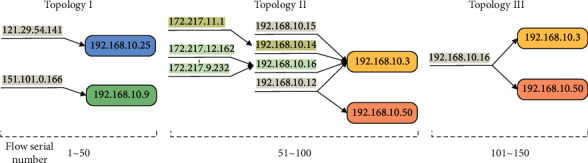
Example of topology structure changing in CIC-IDS2017.

Commonly used cybersecurity datasets include NSL-KDD2009 [54], CIC-IDS2017 [55], CIC-IDS2018 [56], and CIC-DDoS2019 [57]. In particular, CIC-IDS2018 and CIC-DDoS2019 simulate DDoS attacks through point-to-point transmission and lack topology characterization, which cannot meet the needs of this study. In addition to the 76 basic features collected based on CICFlowMeter, CIC-IDS2017 includes the timestamp, source IP and port, destination IP and port, protocol, and attack type. Topology structures in different periods can be obtained through the connection relationship's change between the source and destination IPs. Although IPs are not added in NSL-KDD2009, they can be obtained by parsing the original pcap of DARPA 98 and associated with the traffic record. Therefore, this study chooses two public datasets, NSL-KDD2009 and CIC-IDS2017, as the experimental datasets. The attack on the fifth day of CIC-IDS2017 was a DDoS attack, and its traffic was recorded in “Friday-WorkingHours-Afternoon-DDos.pcap_ISCX.csv.” The topology change is illustrated by taking every 50 traffic records of the fifth day as a unit. Further, four destination IPs, 192.168.10.25/3/50/9, are selected as examples, as shown in Figure 6.

In Figure 6, a typical DDoS topology A-B-C appears in period 51∼100. Compared with the other two periods, the topology has changed significantly. From the number, there is a jump change 2-3-2; from the structure, there are different connection relationships: one-to-one, many-to-one, and one-to-many. The connections with the same source or destination address at various stages are also distinct. In brief, the topological changes like Figure 6 in the dataset can support the validation of the findings of this study.

The first dataset, NSL-KDD2009, is an improved version of KDD99 [58]. It optimizes some inherent problems of KDD99, such as the repeated identical records, missing data, and disproportionate training and testing data. This dataset covers 39 conventional attack methods, including six information gathering (probe), ten blocking attacks (DDoS), nine privilege acquisition (U2L), and 14 remote logins (R2L). Its traffic composition is shown in Table 2, where the bold characters indicate the attack types of the training data.

The second dataset, CIC-IDS2017, was developed by Sharafaldin et al. [59] to implement real network traffic collection based on user behavior simulation. It optimizes NSL-KDD2009 by adding the latest attack methods, expanding the feature set, and adding metadata. Fourteen new attack methods are included: two information gathering, six DDoS attacks, three privilege acquisition, and three remote logins. The dataset is not differentiated by training and test data but by acquisition period.  Table 3 describes its composition.

### 4.3. Preprocessing

DDoS-related traffic records are extracted from NSL-KDD2009 and CIC-IDS2017 and classified according to different attack principles, as shown in Table 4. Due to erroneous data, improper formatting, and redundancy, these two datasets require preprocessing before neural network training.

First, non-numeric features are standardized. There are two types of non-numeric features in the dataset: irrelevant and categorical strings. The former, such as Flow ID, Source IP/Port, Destination IP/Port, and Timestamp in CIC-IDS2017, have nothing to do with flow characterization and are removed from the dataset. For the latter, such as protocol_type, service, and flag in NSL-KDD2009, its classification includes detection information, which must be converted before use. There are two standard methods of string conversion: one-hot and normalized encoding [60]. One-hot sparse matrix has an enormous dimension and low computational efficiency. This study uses continuous integers [0,1,2, ...] to encode the classification and map it between zero and one through normalization.

Secondly, the classification labels are digitized. Unlike categorical features, the Euclidean distance between labels used for error metrics cannot be represented by consecutive integers with uneven differences. After one-hot encoding, the distance between categories is the same and easy to matrix transformation. Therefore, we choose one hot to represent labels for efficient loss function computation. Then, the labels translate to normal traffic (1,0,0), fast traffic (0,1,0), and slow traffic (0,0,1). The columns marked with one here represent the corresponding classifications.

Thirdly, the invalid data are removed. There are two types of useless data: useless row or column data. Useless row data include rows containing ambiguous characters “NaN” and “Infinity.” Useless column data include the column where the 43rd feature “success_pred” of NSL-KDD2009 is located. This feature denotes the number of correct predictions, regardless of traffic attributes. Both are deleted from the dataset directly.

Finally, topological features are extracted and normalized. According to Algorithm 1, the topological structure data are obtained. We use min-max to normalize features to cancel the influence of different scales. *x*_min_ and *x*_max_ are used to represent the minimum and maximum values of feature *x*, respectively, and its calculation formula is as follows:

(14) $$\begin{array}{c} \tilde{x}=\frac{x-x_{\min}}{x_{\max}-x_{\min}}. \end{array}$$

After the above processing, NSL-KDD2009 is transformed into a matrix consisting of 41 features and 45927 moments. CIC-IDS2017 is transformed into a matrix composed of 77 elements, with a total of 2827876 moments. The values in each matrix are between 0 and 1; then, we get normalized input data that are easy for deep learning architectures to process.

### 4.4. Performance Metrics

The detection accuracy verification of the proposed method includes distinguishing between background traffic and attack traffic and between regular traffic, fast attack traffic, and slow attack traffic. The former is a two-class problem, and the latter is a three-class problem. The two have different fine-grained partitions, so we use the targeted evaluation criteria to measure. For binary classification, the indicators are established through the confusion matrix, which has four essential components: true positive (TP), false positive (FP), true negative (TN), and false negative (FN). TP refers to the correct classification of positive samples as positive classes; FP is the proportion of negative samples misidentified as positive classes; TN refers to the correct classification of negative samples as negative classes; and FN refers to the misclassification of positive samples as negative classes. Based on the combination of these parts, we can get four performance metrics: precision, recall, precision, and F1-score. In particular, accuracy is the proportion of correctly classified samples *x*_*correct*_ to the total samples *x*_*total*_. The formula can be expressed as follows:

(15) $$\begin{array}{c} accuracy=\frac{x_{correct}}{x_{total}}=\frac{TP+TN}{TP+TN+FP+FN}. \end{array}$$

Recall is the ratio of correctly classified positive samples ${\bar{x}}_{correct}$ to the total positive samples ${\bar{x}}_{total}$. Its calculation is as follows:

(16) $$\begin{array}{c} recall=\frac{{\bar{x}}_{correct}}{{\bar{x}}_{total}}=\frac{TP}{TP+FN}. \end{array}$$

Precision is the ratio of the correctly classified positive samples ${\bar{x}}_{correct}$ to the detected positive samples *x*_*total*^+^_. The formula is expressed as follows:

(17) $$\begin{array}{c} precision=\frac{{\bar{x}}_{correct}}{x_{total^{+}}}=\frac{TP}{TP+FP}. \end{array}$$

F1-score refers to the weighted harmonic mean of recall and precision. It is used to measure the relative stability of the two. Its formula is as follows:

(18) $$\begin{array}{c} F1-\mathrm{score}={\left(\frac{{\text{recall}}^{-1}+{\text{precision}}^{-1}}{2}\right)}^{-1}=\frac{2TP}{2TP+FP+FN}. \end{array}$$

For triple classification, we adopt comprehensive metrics to measure the overall performance of the detection method, such as macro-average and micro-average. In particular, the macro-average calculates the mean of the metrics under all categories; the micro-average is an extension of the two-category metrics. Considering all class effects, we choose the macro-average as the three-class measure. Let *n* denote the number of classifications and *Xi* denote the *i*th value of indicator *X*, and the formula is as follows:

(19) $$\begin{array}{c} \text{Macro}\_X=\frac{1}{n}\sum_{i=1}^{n}X_{i}. \end{array}$$

Furthermore, this study also studies the relationship between the evaluation result and the distribution of attack source IPs. The Pearson coefficient is used as the correlation measure. Let cov(*X*, *Y*), *σ*_*x*_, and *σ*_*y*_ denote the formulas for calculating the covariance and variance of variables *x* and *y*, respectively, and *E*(*X*) and *E*(*Y*) denote the expectation calculation. Its formula is as follows:

(20) $$\begin{array}{c} \rho \left(x,y\right)=\frac{\mathrm{cov}\left(X,Y\right)}{\sigma_{x}\sigma_{y}}=\frac{E\left(XY\right)-E\left(X\right)E\left(Y\right)}{\sqrt{E\left(X^{2}\right)-E^{2}\left(X\right)}\sqrt{E\left(Y^{2}\right)-E^{2}\left(Y\right)}}. \end{array}$$

### 4.5. Results and Analysis

As shown in Table 4, the traffic composition of NSL-KDD2009 and CIC-IDS2017 is quite different. For these imbalanced datasets, 10-fold cross-validation is used for optimization. This method divides the sample into ten equal subsamples, sequentially uses one part for testing and the remaining nine parts for training, and takes the average of 10 times as the final result.

Testing the same method on different datasets may yield different results. Therefore, all DDoS detection methods are validated on the same dataset for comparative effectiveness. In addition, the same features in Table 1 were chosen for training for control variables.

The experiment consists of four parts: the comparison of two-classification methods, the comparison of three-classification methods, the correlation analysis of source IP distribution, and the analysis of method performance. Two-classification and three-classification methods are not always the same. Therefore, different baselines and state-of-the-art methods are selected as the comparison objects for the two comparison experiments.

#### 4.5.1. Two-Classification Experiment

We choose six baseline and state-of-the-art methods as comparison objects in the binary classification experiment. In particular, baselines include the statistical method NaHiD [22], machine learning SKM-HFS [25], and random forest [26]; the latest methods include LUCID [30], DDoSNet [9], and BI-LSTM-GMM [34]. Baselines are reproduced with the Python library. In particular, NaHiD is obtained according to the mean and standard deviation of NumPy. SKM-HFS and random forest are calculated according to scikit-learn. These methods do not support GPU, so all baselines run on CPU. Furthermore, deep learning runs on GPU and compares the efficiency.

**Figure 7:**
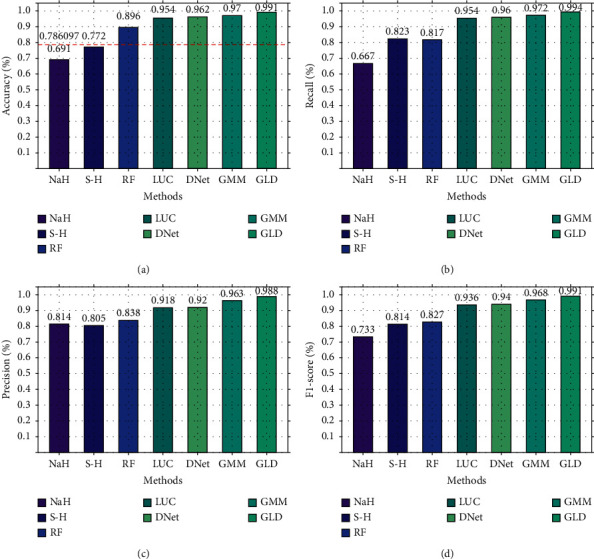
Comparison of binary classification performance between GLD-Net and other six methods on NSL-KDD2009.

First, on NSL-KDD2009, GLD-Net is compared with six other means to verify binary classification performance. The epochs are set to 100, and the results are shown in Figure 7. As shown in Figure 7, these methods have different effects. Random forest achieves the best performance among the baselines with an accuracy of 0.896. The three deep learning methods show better detection performance with scores all above 0.9. In particular, BI-LSTM-GMM achieved the highest accuracy of 0.97 among the three. GLD-Net performs the highest metric on four indicators compared with the above techniques. Its accuracy reaches 0.991, which is 0.205 and 0.021 higher than the baselines and BI-LSTM-GMM, respectively.

**Figure 8:**
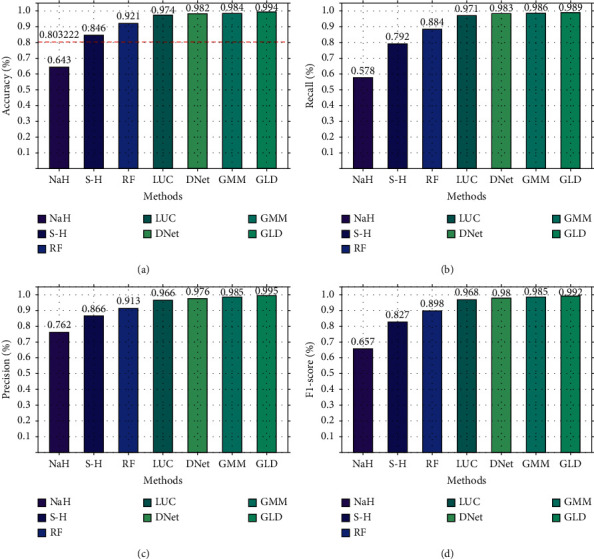
Comparison of binary classification performance between GLD-Net and other six methods on CIC-IDS2017.

Secondly, we also conducted a binary classification comparison experiment on CIC-IDS2017, and the result is shown in Figure 8. As shown in Figure 8, there is a significant gap between different methods. NaHiD still performs poorly, with both precision and recall not exceeding 0.65. The accuracy of deep learning is excellent, all exceeding 0.95. Compared with the other six methods, GLD-Net achieves the best performance, 0.191 and 0.0101 higher than the baseline and BI-LSTM-GMM.

**Figure 9:**
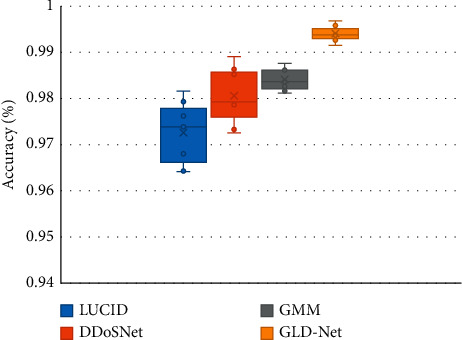
Performance distribution comparison between GLD-Net and three state-of-the-art methods.

Finally, we compare the accuracy distributions of GLD-Net and the three newest methods on two classifications, as shown in Figure 9. From Figure 9, GLD-Net has the highest accuracy and a concentrated distribution across multiple tests. The upper and lower quartile distances of LUCID and DDoSNet exceed 0.01, and the gap between the maximum and the minimum is close to 0.02. In contrast, the quantile distance of GLD-Net is only 0.003, more than four times lower than the average distance of 0.014 of other newest methods, showing the stability of the attack detection.

In summary, the accuracy of GLD-Net on NSL-KDD2009 and CIC-IDS2017 reaches 0.9914 and 0.9942, respectively. Compared with the latest methods, its average improvements are 0.021 and 0.0101; its stability increases four times, showing the best detection performance and stability.

#### 4.5.2. Three-Classification Experiment

Given the low precision, statistical methods are usually not used to solve multi-classification problems. We choose five baselines and state-of-the-art methods as comparison objects in three-classification experiments. Among them, the baseline methods include decision tree [27] and random forest [26], and the latest methods include Stacked-DNN [36], FastGRNN [37], and ResNet [38]. Baselines are calculated according to scikit-learn.

**Figure 10:**
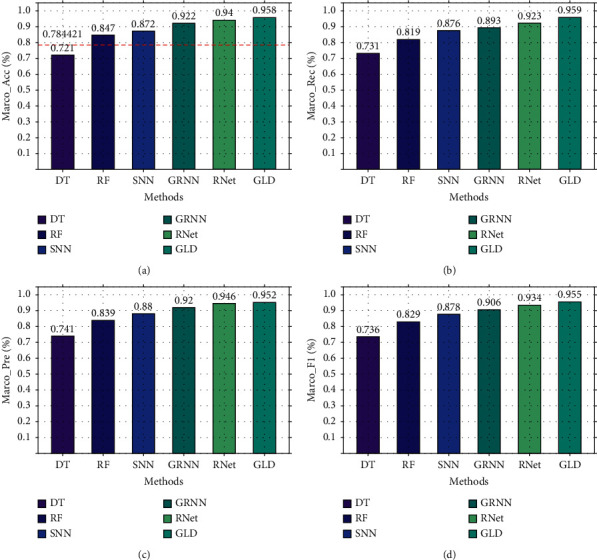
Three-class performance comparison of GLD-Net and other five methods on NSL-KDD2009.

First, we compare the three-classification performance of GLD-Net and five other methods on NSL-KDD2009. The results are shown in Figure 10. As shown in Figure 10, GLD-Net achieves the best three-classification performance compared with other methods. Its macro-accuracy reaches 0.958, an average improvement of 0.174 and 0.047 over baselines and the newest techniques.

**Figure 11:**
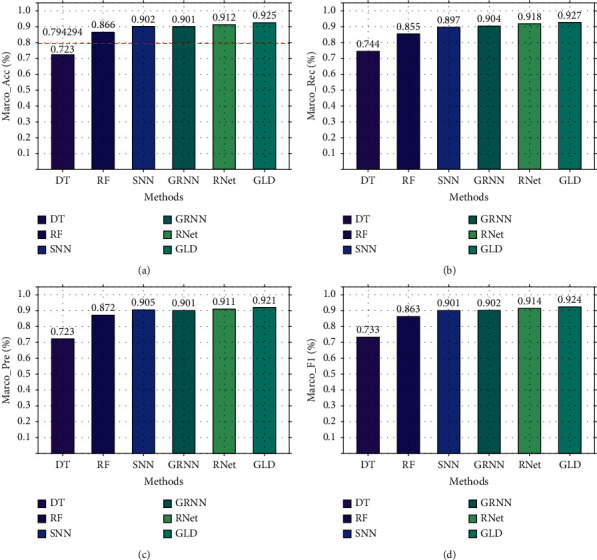
Three-class performance comparison of GLD-Net and other five methods on CIC-IDS2017.

Secondly, based on CIC-IDS2017, the three-classification experiment was performed, and the results are shown in Figure 11. As shown in Figure 11, GLD-Net achieves the best performance, with the accuracy and F1-score reaching 0.925 and 0.924, respectively. Compared to Figure 10, deep learning performance degrades partly due to varying traffic types in datasets. Compared with baselines and the newest methods' averages, GLD-Net improves the accuracy by 0.131 and 0.019, respectively.

**Figure 12:**
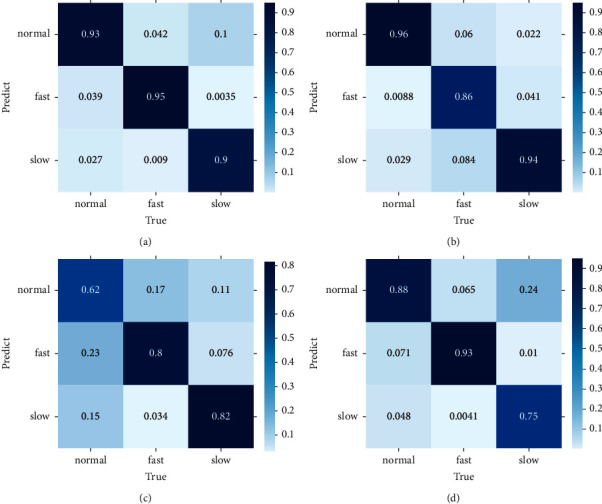
Confusion matrix of GLD-Net and three other methods for three-class detection. (a) GLD-Net. (b) ResNet. (c) Decision tree. (d) Random forest.

Finally, we compare the difference in the confusion matrix among GLD-Net and three other methods, as shown in Figure 12. The colors in the graph range from white to blue, representing accuracy from 0 to 1.0. The darker the blue, the higher the ratio. As shown in Figure 12, GLD-Net achieves good results in traffic type detection, and TP exceeds 0.9. While the normal flow of decision tree, slow flow of random forest, and fast flow of ResNet have lower TP, which is 0.62, 0.75, and 0.86, respectively. Detection based on GLD-Net has better balance and can meet the needs of fine-grained discrimination.

Compared with the state-of-the-art methods, the three-class accuracy of GLD-Net is improved by 0.047 and 0.02, respectively. Its availability is also increased by 0.023, showing better performance and broad applicability.

#### 4.5.3. Distribution Correlation Analysis

**Figure 13:**
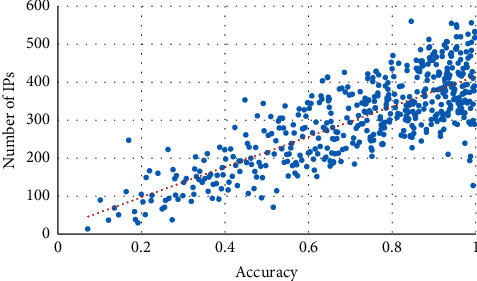
Correlation between TP and IP number under GLD-Net.

First, the correlation between TP of attack detection and the number of attack source IPs is investigated. The results are shown in Figure 13. It can be seen from Figure 13 that there is a positive correlation between the TP of GLD-Net and the number of attack source IPs. Its Pearson coefficient is 0.789; greater than 0.75 shows a strong correlation.

**Figure 14:**
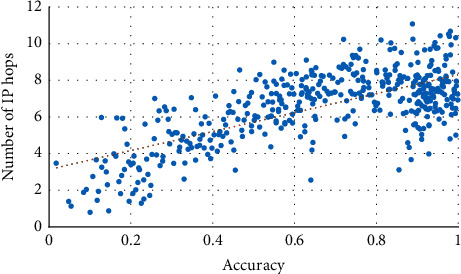
Correlation between TP and IP hop count under GLD-Net.

Secondly, the correlation between TP and the IP hop count (the average hop count of all leaf nodes) is examined. The result is shown in Figure 14. As shown in Figure 14, a positive correlation exists between TP and IP hops' numbers using GLD-Net. The Pearson coefficient is 0.695, close to 0.7, indicating that the two have specific relevance.

**Figure 15:**
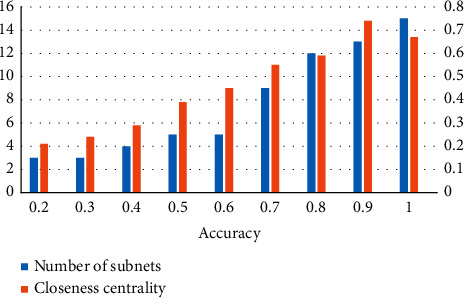
Variation trend of detection TP, number of subnets, and closeness centrality of GLD-Net.

Thirdly, at the network level, the number of subnets [61] and closeness centrality [62] are used to investigate the aggregation and distribution of attack sources. The results are shown in Figure 15. It can be seen from Figure 15 that the number of subnetworks and closeness centrality increase with the rise of TP. After 200 cycles of calculation and taking the mean value, the Pearson coefficients obtained are 0.812 and 0.834, respectively, and over 0.8 indicates a strong correlation.

**Figure 16:**
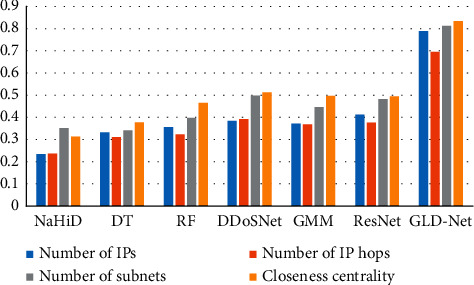
Comparison of correlation coefficients between GLD-Net and other six methods for detecting TP and four distribution metrics.

Finally, GLD-Net is compared with other detection methods using non-topological features as input in the correlation between TP and attack source IP number, hop number, subnet number, and closeness centrality. The results are shown in Figure 16. It can be seen from Figure 16 that the correlation coefficients of the comparative methods are primarily in the range 0.3∼0.5. The correlation coefficients of GLD-Net fall on the interval 0.7∼0.83, and the average increases in the four correlations are 0.441, 0.36, 0.393, and 0.391, respectively. The rise of around 0.4 shows that GLD-Net is more capable of inferring the distribution of attack sources based on the detection results than other methods.

In short, we found that the four attack source distribution indicators have correlation coefficients with TP of GLD-Net reaching 0.789, 0.695, 0.812, and 0.834, respectively. Compared with other methods that take non-topological features as input, the average improvement is 0.441, 0.36, 0.393, and 0.391, respectively. The strong correlation of 0.7∼0.83 supports using the evaluation result to infer the distribution of attack sources. For example, when TP is 0.8, combined with Figures 13–15, it can be deduced that the attack source IPs' number is around 350, hops' number is 7, subnets' number is 12, and closeness centrality is 0.59.

#### 4.5.4. Efficiency Analysis

**Figure 17:**
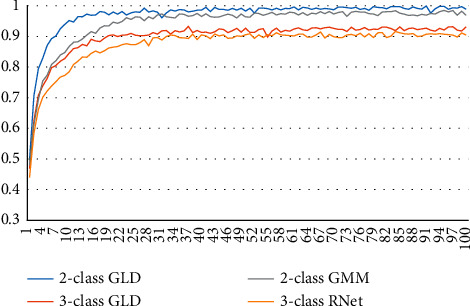
Comparison of accuracy trends of GLD-Net and other methods on two-classification and three-classification detection during 100 epoch training.

First, we investigate the accuracy variation of GLD-Net during 100 epochs. We choose BI-LSTM-GMM and ResNet as the two-classification and three-classification comparison objects. The results are shown in Figure 17. It can be seen from Figure 17 that the accuracy of BI-LSTM-GMM and ResNet tends to be stable at the 22nd and 28th epochs, respectively. The accuracy of GLD-Net gradually stabilized at the 11th or 16th epoch. In contrast, our proposed model can converge faster and achieve better performance.

**Figure 18:**
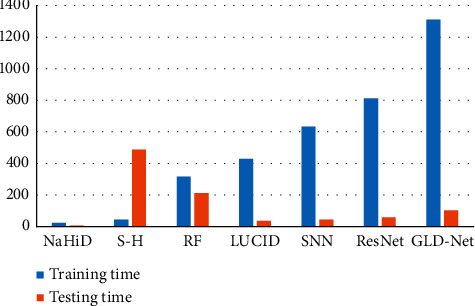
Comparison of training and testing times for GLD-Net and other six standard methods.

Secondly, we also analyze the training and testing time of GLD-Net. We selected six other methods for comparison, and the result is shown in Figure 18. As shown in Figure 18, the average training time of GLD-Net reaches 1312 s, 4.14 times that of random forest and 1.59 times that of ResNet. The test time of GLD-Net is 107 s, which is 8.16% of its training time and 50.47% of random forest. The results show that although the training time is slightly longer than other deep learning methods, the test time is still within the tolerance range. This overhead is worthwhile compared with the improved accuracy.

To summarize, GLD-Net converges in 11 or 16 epochs in binary or multi-classification, an average of 11.5 epochs ahead of the best other methods. The average training and testing times of GLD-Net are 1312 s and 107 s, respectively. Its training time is 4.14 times that of random forest; the test time is only 50.47% of that of random forest, indicating the better practical efficiency of GLD-Net.

## 5. Conclusions

In this study, we propose GLD-Net, a new deep learning DDoS attack detection method based on topological and flow features. A graph model is introduced for feature extraction. Traffic features are added to edge features, and node features represent topological features. A dynamic DDoS topology feature construction algorithm is proposed by calculating the feature table and mapping topological entities on the time series. For non-Euclidean input, GAT mines complex topological relationships, and LSTM extracts sequence correlation in vectors. Finally, the fully connected layer obtains the traffic type through data integration. The experimental results show that DDoS detection with topology and flow features as input can solve the problem of limited accuracy due to incomplete feature input. It can also estimate the distribution of attack sources based on the detection results, which facilitates the rapid and accurate deployment of subsequent security strategies. In the future, we also need to design a more fine-grained differentiation model for different traffic types and explore unknown traffic discovery techniques. These related researches will expand the scope of application to escort system security in the current increasingly complex network confrontation situation.

## 6. Future Research

We verified the effectiveness of the proposed DDoS detection method GLD-Net through comparative experiments on two network security datasets. Nevertheless, there are still the following issues to be studied.

**Question 1 .**

We mainly verify the method's performance in distinguishing normal, fast, and slow traffic for the multi-classification. Whether this method is suitable for more fine-grained differentiation, such as HTTP applications, requires further verification.

**Question 2 .**

The neighborhood computation efficiency of GAT is not high, and it cannot cope with real-time training. Next, we need to study a lightweight GAT structure to improve the execution speed.

**Question 3 .**

This research mainly focuses on relationship mining in the existing traffic. It cannot discover unknown traffic such as 0-day attacks. Therefore, it is necessary to study novel deep learning methods that simultaneously identify known and anonymous traffic.

## Figures and Tables

**Algorithm 1:**
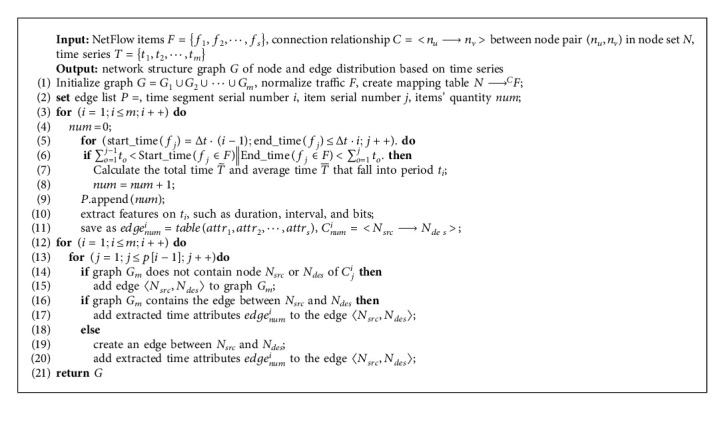
Construction of dynamic DDoS topology graph based on time series.

**Table 1:** Node or edge TCP features collected from time series.

Item Object
	Name	Type	Number	Description
Edge	Connection number	Integer	1	Total number of traffic records in *t*_s_
	Connection states	List	States' number	Number of communication states in *t*_s_
	Duration	Float	6	Total, mean, median, standard deviation, maximum, and minimum of flow duration
	Packet interval	Float	6	Total, mean, median, standard deviation, maximum, and minimum of the packets' interval
	Forward packets' number	Float	6	Total, mean, median, standard deviation, maximum, and minimum of the forward packets' number
	Backward packets' number	Float	6	Total, mean, median, standard deviation, maximum, and minimum of the backward packets' number
	URG flag	List	Packets' number	Sequences of URG flags in *t*_s_
	ECE flag	List	Packets' number	Sequences of ECE flags in *t*_s_

Node	Degree centrality	Float	1	Number of neighbor nodes connected to the node within *t*_s_
	Betweenness centrality	Float	1	Number of shortest paths passing through the node within *t*_s_

**Table 2:** Traffic composition of the NSL-KDD2009 dataset.

Type	Attack method
Benign	normal
Probe	**ipsweep**, mscan, **nmap**, **portsweep**, saint, **satan**
DDoS	apache2, **back**, **land**, mailbomb, **neptune**, **pod**, processtable, **smurf**, **teardrop**, udpstrom
U2L	**bufferoverflow**, **loadmodule**, **perl**, ps, **rootkit**, snmpguess, sqlattack, worm, xterm
R2L	**ftp_write**, **guesspasswd**, httptunnel, **imap**, **multihop**, named, **phf**, **spy**, sendmail, snmpgetattack, **warezclient**, **warezmaster**, xlock

**Table 3:** Traffic composition of the CIC-IDS2017 dataset.

Type	Attack method
Benign	BENIGN
Probe	Heartbleed, PortScan
DDoS	DoS Hulk, DDoS, DoS GoldEye, DoS slowloris, DoS Slowhttptest, bot
U2L	Web Attack-XSS, Infiltration, Web Attack-Sql Injection
R2L	FTP-Patator, SSH-Patator, Web Attack-Brute Force

**Table 4:** Number and classification of DDoS-related traffic records.

Datasets	Attack method	Sort	Number	Total
NSL-KDD 2009	0. normal	Normal 0	67,343	113,270
	1. neptune	Fast flow (1,2,3)	41,214	
	2. pod		201	
	3. smurf		2,646	
	4. back	Slow flow (4,5,6)	956	
	5. land		18	
	6. teardrop		892	

CIC-IDS 2017	0. BENIGN	Normal 0	2,273,097	2,655,751
	1. bot	Fast flow (1,2,3,4)	1,966	
	2. DoS Hulk		231,073	
	3. DDoS		128,027	
	4. DoS GoldEye		10,293	
	5. DoS slowloris	Slow flow (5,6)	5,796	
	6. DoS Slowhttptest		5,499	

## Data Availability

The NSL-KDD2009 and CIC-IDS2017 datasets used to support the finding of this study are included within the article.

## References

[1] Abusaimeh H. (2020). Distributed denial of service attacks in cloud computing. *International Journal of Advanced Computer Science and Applications*.

[2] Agarwal A., Khari M., Singh R. (2021). Detection of DDOS Attack Using Deep Learning Model in Cloud Storage Application. *Wireless Personal Communications*.

[3] Alatawi F. (2021). Defense mechanisms against distributed denial of service attacks: comparative review. *Journal of Information Security and Cybercrimes Research*.

[4] Wu Z., Wei Q., Ren K., Wang Q. (2017). Dynamic defense for DDoS attack using openflow-based switch shuffling approach. *Dianzi Yu Xinxi Xuebao/Journal of Electronics and Information Technology*.

[5] Singh K., Singh Dhindsa K., Bhushan B. (2017). Distributed Defense: An Edge over Centralized Defense against DDos Attacks. *International Journal of Computer Network and Information Security*.

[6] Liu Y., Zhi T., Shen M., Wang L., Li Y., Wan M. Software-defined DDoS detection with information entropy analysis and optimized deep learning. *Future Generation Computer Systems*.

[7] Mittal M., Kumar K., Behal S. (2022). Deep Learning Approaches for Detecting DDoS Attacks: A Systematic Review. *Soft Computing*.

[8] Pektaş A., Acarman T. (2019). Deep learning to detect botnet via network flow summaries. *Neural Computing & Applications*.

[9] Elsayed M. S., Le-Khac N. A., Dev S., Jurcut A. D. *DDoSNet: A Deep-Learning Model for Detecting Network Attacks*.

[10] He J., Tan Y., Guo W., Xian M. A Small Sample DDoS Attack Detection Method Based on Deep Transfer Learning.

[11] Liaskos C., Ioannidis S. (2018). Network topology effects on the detectability of crossfire attacks. *IEEE Transactions on Information Forensics and Security*.

[12] Sharma K., Mukhopadhyay A. (2021). Kernel naïve Bayes classifier-based cyber-risk assessment and mitigation framework for online gaming platforms. *Journal of Organizational Computing & Electronic Commerce*.

[13] Shafi Q., Basit A. DDoS Botnet Prevention Using Blockchain in Software Defined Internet of Things.

[14] Veličković P., Casanova A., Liò P., Cucurull G., Romero A., Bengio Y. (2018). Graph Attention Networks. https://arxiv.org/abs/1710.10903.

[15] Zhang C., Cheng J., Tang X., S Sheng V., Dong Z., Li J. (2019). Novel DDoS feature representation model combining deep belief network and canonical correlation analysis. *Computers, Materials & Continua*.

[16] Cui Y., Qian Q., Guo C. Towards DDoS detection mechanisms in Software-Defined Networking. *Journal of Network and Computer Applications*.

[17] Yuan X., Li C., Li X. DeepDefense: Identifying DDoS Attack via Deep Learning.

[18] Idhammad M., Afdel K., Belouch M. (2018). Semi-supervised machine learning approach for DDoS detection. *Applied Intelligence*.

[19] Doshi R., Apthorpe N., Feamster N. Machine Learning DDoS Detection for Consumer Internet of Things Devices.

[20] De Lima Filho F. S., Silveira F. A. F., De Medeiros Brito Junior A., Vargas-Solar G., Silveira L. F. (2019). Smart Detection: An Online Approach for DoS/DDoS Attack Detection Using Machine Learning. *Security and Communication Networks*.

[21] Chouhan R. K., Atulkar M., Nagwani N. K. (2022). A Framework to Detect DDoS Attack in Ryu Controller Based Software Defined Networks Using Feature Extraction and Classification. *Applied Intelligence*.

[22] Hoque N., Kashyap H., Bhattacharyya D. K. (2017). Real-time DDoS attack detection using FPGA. *Computer Communications*.

[23] Tsobdjou L. D., Pierre S., Quintero A. (2022). An Online Entropy-Based DDoS Flooding Attack Detection System with Dynamic Threshold. *IEEE Transactions on Network and Service Management*.

[24] Ahalawat A., Babu K. S., Turuk A. K., Patel S. (2022). A low-rate DDoS detection and mitigation for SDN using Renyi Entropy with Packet Drop. *Journal of Information Security and Applications*.

[25] Gu Y., Li K., Guo Z., Wang Y. (2019). Semi-supervised k-means ddos detection method using hybrid feature selection algorithm. *IEEE Access*.

[26] Pande S., Khamparia A., Gupta D., Thanh D. N. H. (2021). DDOS detection using machine learning technique. *Studies in Computational Intelligence*.

[27] Cvitic I., Perakovic D., Gupta B. B., Choo K. K. R. (2022). Boosting-based DDoS detection in Internet of things systems. *IEEE Internet of Things Journal*.

[28] Kumar S., G Sastry H., Marriboyina V. (2022). Ddos detection in sdn usingmachine learning techniques. *Computers, Materials & Continua*.

[29] Liang X., Znati T. A Long Short-Term Memory Enabled Framework for DDoS Detection.

[30] Doriguzzi-Corin R., Millar S., Scott-Hayward S., Martinez-Del-Rincon J., Siracusa D. (2020). Lucid: a practical, lightweight deep learning solution for DDoS attack detection. *IEEE Transactions on Network and Service Management*.

[31] Cil A. E., Yildiz K., Buldu A. Detection of DDoS attacks with feed forward based deep neural network model. *Expert Systems with Applications*.

[32] Boonchai J., Kitchat K., Nonsiri S. The classification of DDoS attacks using deep learning techniques.

[33] Wang L., Liu Y. A DDoS Attack Detection Method Based on Information Entropy and Deep Learning in SDN.

[34] Shieh C. S., Lin W. W., Nguyen T. T., Chen C. H., Horng M. F., Miu D. (2021). Detection of unknown ddos attacks with deep learning and Gaussian mixture model. *Applied Sciences*.

[35] Almaraz-Rivera J. G., Perez-Diaz J. A., Cantoral-Ceballos J. A. (2022). Transport and application layer DDoS attacks detection to IoT devices by using machine learning and deep learning models. *Sensors*.

[36] Toupas P., Chamou D., Giannoutakis K. M., Drosou A., Tzovaras D. An Intrusion Detection System for Multi-Class Classification Based on Deep Neural Networks.

[37] Alzahrani H., Abulkhair M., Alkayal E. (2020). A multi-class neural network model for rapid detection of IoT botnet attacks. *International Journal of Advanced Computer Science and Applications*.

[38] Hussain F., Abbas S. G., Husnain M., Fayyaz U. U., Shahzad F., Shah G. A. IoT DoS and DDoS Attack Detection Using ResNet.

[39] Rusyaidi M., Jaf S., Ibrahim Z. (2022). Detecting distributed denial of service in network traffic with deep learning. *International Journal of Advanced Computer Science and Applications*.

[40] Novaes M. P., Carvalho L. F., Lloret J., Proença M. L. (2021). Adversarial Deep Learning approach detection and defense against DDoS attacks in SDN environments. *Future Generation Computer Systems*.

[41] Doriguzzi-Corin R., Siracusa D. (2022). FLAD: adaptive federated learning for DDoS attack detection. https://arxiv.org/abs/2205.06661.

[42] Lohachab A., Karambir B. (2018). Critical analysis of DDoS—an emerging security threat over IoT networks. *Journal of Communications and Information Networks*.

[43] Kousar H., Mulla M. M., Shettar P., Narayan D. G. Detection of DDoS Attacks in Software Defined Network Using Decision Tree.

[44] Liang J., Chen J., Zhang X., Zhou Y., Lin J. (2019). One-hot encoding and convolutional neural network based anomaly detection. *Qinghua Daxue Xuebao/Journal of Tsinghua University*.

[45] Abdulrahman A. A., Ibrahem M. K. (2019). Evaluation of DDoS attacks detection in a new intrusion dataset based on classification algorithms. *Iraqi Journal of Information & Communications Technology*.

[46] Tang X., Cao R., Cheng J., Fan D., Tu W. (2019). DDoS attack detection method based on V-Support vector machine. *Lecture Notes in Computer Science (including subseries Lecture Notes in Artificial Intelligence and Lecture Notes in Bioinformatics)*.

[47] Kasim Ö. (2020). An efficient and robust deep learning based network anomaly detection against distributed denial of service attacks. *Computer Networks*.

[48] Grassia M., De Domenico M., Mangioni G. (2021). Machine learning dismantling and early-warning signals of disintegration in complex systems. *Nature Communications*.

[49] Tayfour O. E., Marsono M. N. (2021). Collaborative detection and mitigation of DDoS in software-defined networks. *The Journal of Supercomputing*.

[50] Zhang X., Zou Y., Shi W. Dilated convolution neural network with LeakyReLU for environmental sound classification.

[51] Sherstinsky A. (2020). Fundamentals of recurrent neural network (RNN) and long short-term memory (LSTM) network. *Physica D: Nonlinear Phenomena*.

[52] Huang X., Tan H., Lin G., Tian Y. A LSTM-Based Bidirectional Translation Model for Optimizing Rare Words and Terminologies.

[53] Zhang L., Shi Z., Cheng M. M. (2021). Nonlinear regression via deep negative correlation learning. *IEEE Transactions on Pattern Analysis and Machine Intelligence*.

[54] Dhanabal L., Shantharajah S. P. (2015). A study on NSL-KDD dataset for intrusion detection system based on classification algorithms. *International Journal of Advanced Research in Computer and Communication Engineering*.

[55] Zhou Y., Cheng G., Jiang S., Dai M. Building an efficient intrusion detection system based on feature selection and ensemble classifier. *Computer Networks*.

[56] Leevy J. L., Khoshgoftaar T. M. (2020). A survey and analysis of intrusion detection models based on CSE-CIC-IDS2018 Big Data. *Journal of Big Data*.

[57] Sharafaldin I., Lashkari A. H., Hakak S., Ghorbani A. A. Developing realistic distributed denial of service (DDoS) attack dataset and taxonomy.

[58] Tavallaee M., Bagheri E., Lu W., Ghorbani A. A. A Detailed Analysis of the KDD CUP 99 Data Set.

[59] Sharafaldin I., Lashkari A. H., Ghorbani A. A. Toward generating a new intrusion detection dataset and intrusion traffic characterization.

[60] Rodríguez P., Bautista M. A., Gonzàlez J., Escalera S. (2018). Beyond one-hot encoding: lower dimensional target embedding. *Image and Vision Computing*.

[61] Dimolianis M., Pavlidis A., Maglaris V. A Multi-Feature DDoS Detection Schema on P4 Network Hardware.

[62] Li J., Lyu L., Liu X., Zhang X., Lyu X. (2022). FLEAM: a federated learning empowered architecture to mitigate DDoS in industrial IoT. *IEEE Transactions on Industrial Informatics*.

